# Stratum corneum hydration inversely correlates with certain serum cytokine levels in the elderly, possibly contributing to inflammaging

**DOI:** 10.1186/s12979-023-00331-1

**Published:** 2023-02-07

**Authors:** Bin Yang, Chengzhi Lv, Li Ye, Zhen Wang, Yoon Kim, Wenhai Luo, Peter M. Elias, Mao-Qiang Man

**Affiliations:** 1 Dermatology Hospital, Southern Medical University, 2 Lujing Road, Guangzhou, 510091 Guangdong China; 2 Department of Dermatology, Dalian Skin Disease Hospital, Liaoning, 116021 China; 3 The 7Th People’s Hospital of Shenyang, Liaoning, 110003 China; 4 CRID Center, NeoPharm Co., Ltd, Daejeon, 34037 Republic of Korea; 5 School of Public Health and Management, Binzhou Medical University, Shandong, 264003 China; 6 Deparmtnet of Dermatology, University of California San Francisco and Veterans Affairs Medical Center, San Francisco, CA 94121 USA

**Keywords:** Aging, pH, Hydration, Barrier, Epidermis, Inflammation, Inflammaging, Cytokines

## Abstract

**Background:**

Chronic, low-grade inflammation, also termed ‘inflammaging’, has been linked to the development of some aging-associated disorders. Recent studies suggest that inflammaging is attributable to aging-associated epidermal dysfunction. However, abnormality in which epidermal function contributes to inflammaging is not clear.

**Objective:**

We delineated the correlation of epidermal functions with circulating levels of proinflammatory cytokines in the elderly.

**Methods:**

Blood sample was collected from a total of 255 participants aged ≥ 65 years. Epidermal biophysical properties were measured on the left forearm and the right shin. Serum cytokine levels were measured by Multiplex Luminex Assays.

**Results:**

Neither skin surface pH nor transepidermal water loss rates (TEWL) correlated with serum cytokine levels except TEWL on the right shin for TNFa (*p* < 0.05). In contrast, stratum corneum hydration levels on both the forearm and the shin correlated negatively with serum cytokine levels (*p* < 0.05).

**Conclusion:**

Reduced stratum corneum hydration likely contributes to inflammaging.

**To the Editor**

Aged individuals (> 65 years) display chronic, low-grade inflammation [1], termed ‘inflammaging,’ which has been linked to the development of many aging-associated systemic disorders, such as type 2 diabetes, obesity, cardiovascular diseases, cancers, and certain neurodegenerative disorders [2]. Senescent cells, adipose tissue, immune cells, and microbiome dysbiosis have all been widely speculated as being the sources/origins of inflammaging [2], and some or all these speculative sources could contribute to a varying extent. However, one recent line of evidence indicates that epidermal dysfunction contributes to inflammaging. First, chronologically aged individuals exhibit altered epidermal functions, including delayed permeability barrier recovery, reduced levels of stratum corneum hydration, and elevated stratum corneum pH, which all can provoke and/or exacerbate cutaneous inflammation, while sustained cutaneous inflammation in turn can lead to systemic inflammation [3]. Second, improvements in epidermal functions following the applications of topical emollients, such as glycerol, petrolatum, or Atopalm® MLE cream, lowered circulating levels of pro-inflammatory cytokines in both aged mice and aged humans [4, 5]. Glycerol and petrolatum can improve both stratum corneum hydration and epidermal permeability barrier, whereas Atopalm® MLE cream can improve skin surface pH, stratum corneum hydration and the epidermal permeability barrier [5]. Thus, it is not clear which epidermal functional abnormality(ies) is/are linked to inflammaging. To determine which epidermal functional abnormality(ies) contribute(s) to inflammaging, we analyzed the correlation of epidermal function with circulating levels of proinflammatory cytokines in the aged humans.

Following approval by the Institutional Review Board of the Dermatology Hospital, Southern Medical University, China, we performed a retrospective chart review of a previous study on aging in Dalian and Shenyang City, Northern China, in the month of November 2018. Data from a total of 255 subjects, including 83 males and 172 females, aged 65–99 years old, were included in this analysis (detailed in Table 1). Measurements of epidermal biophysical properties were detailed in a previous study [6]. Briefly, epidermal biophysical properties, including transepidermal water loss rates and stratum corneum hydration levels were measured with a GpSkin Barrier (Gpower, inc. Seoul, Korea), while skin surface pH was measured with a skin pH meter pH900 (Courage + Khazaka electronic GmbH, Köln, Germany). The measurement sites were the flexor side of the left forearm, 10 cm above the wrist and the right shin, 15 cm above the ankle. Circulating levels of interferon gamma (IFNgamma), interleukin 1a(IL-1a), IL-1b, IL-6 and tumor necrosis factor (TNF)a were measured using MILLIPLEX MAP Human Cytokine/Chemokine Magnetic Bead Panel (Cat. ID. HCYTOMAG-60 K-05) from Merck, Ltd (Shanghai), according to the manufacturer’s instructions. Graphpad prism 5 was used to determine the correlation of serum cytokine levels with transepidermal water loss (TEWL) rates, skin surface pH and stratum corneum hydration levels.

**Table 1:** Demographic Characteristics and epidermal biophysical properties of subjects

	Males (*N* = 83)	Females (*N* = 172)	Overall (*N* = 255)
Age (Yr) [mean ± sem, IQR, 95%CI]	77.04 ± 0.98, 17, 75.09–78.98	77.34 ± 0.72, 18, 75.94–78.79	77.26 ± 0.58, 18, 76.11–78.40
BMI (mean ± sem, IQR, 95%CI)	23.51 ± 0.32, 3.50, 22.88–24.13	24.55 ± 0.31, 5.86, 23.93–25.17	24.21 ± 0.24, 4.96, 23.74–24.68
SC Hydration (au) on the Forearm	22.89 ± 1.50, 20, 19.91–25.88	18.43 ± 0.90^a^, 17, 16.65–20.21	19.88 ± 0.79, 16, 18.33–21.44
SC Hydration (au) on the Shin	13.49 ± 1.28, 15, 10.94–16.04	11.71 ± 0.74, 9.75, 10.26–13.16	12.29 ± 0.65, 12, 11.01–13.57
TEWL (g/m^2^/hr) on the Forearm	10.88 ± 0.90, 8, 9.097–12.66	11.70 ± 0.97, 10, 9.787–13.62	11.44 ± 0.72, 9, 10.02–12.85
TEWL (g/m^2^/hr) on the Shin	9.64 ± 1.13, 7, 7.400–11.88	8.81 ± 0.68, 7.75, 7.468–10.16	9.08 ± 0.59, 8, 7.926–10.24
Skin Surface pH on the Forearm	5.21 ± 0.07, 0.82, 5.068–5.343	5.51 ± 0.05^b^, 1.02, 5.397–5.612	5.41 ± 0.04, 1.01, 5.321–5.494
Skin Surface pH on the Shin	5.36 ± 0.07, 0.80, 5.126–5.503	5.57 ± 0.05^c^, 0.825, 5.573–5.766	5.57 ± 0.04, 0.96, 5.487–5.650

*IQR:* Interquartile range; *BMI:* Body mass index; *SC:* Stratum corneum; *TEWL:* Transepidermal water loss rate

^a^*p* = 0.0137, ^b^*p* = 0.0011, ^c^*p* = 0.0001 vs. males

Although elevation in skin surface pH is associated with cutaneous inflammation in mice [7], skin surface pH did not correlate positively with the circulating levels of proinflammatory cytokines in this cohort (Table 2). In contrast, serum TNFa levels correlated negatively with skin surface pH in males, suggesting it is unlikely that aging-associated elevation in skin surface pH contributes to inflammaging. Similarly, TEWL rates on neither the forearm nor the shin correlated with proinflammatory cytokine levels in this cohort, except on the right shin for IL-6. After adjusted by age and sex, serum TNFa levels correlated positively with TEWL rates on the shin (*R* = 0.127, *p* = 0.044). On the contrary, reduced stratum corneum hydration levels on both the forearm and the shin correlated negatively with circulating levels of proinflammatory cytokines, especially in females (Table 2). The underlying mechanisms of such gender-related differences in the significance of correlation between stratum corneum levels and serum cytokine levels are not clear. However, significantly lower levels of stratum corneum hydration in females, a determinant of cutaneous and systemic inflammation [3–5, 8], can contribute in part to the gender differences in the significances of the correlation. Moreover, higher BMI in females could also account for the stronger correlation between stratum corneum and serum cytokine levels because BMI is positively associated with inflammation [9]. In addition, the stronger correlation between stratum corneum and serum cytokine levels in females can be attributable to a larger number of subjects in females than in males (172 vs. 83) since generally, the larger the sample size is, the more significant it will be. Notably, TNFa levels correlated positively with age in females (*r* = 0.2250, *p* = 0.003), and both IL-1a (*r* = -0.2375, *p* = 0.0306) and IL-6 levels (*r* = -0.2250, *p* = 0.0408) inversely correlated with age in males. Taken together, these results suggest that increased circulating levels of proinflammatory cytokines in the aged humans are associated with aging-associated declines in stratum corneum hydration.

**Table 2:** Correlation of epidermal biophysical properties with serum cytokine levels

Crude Correlation		Stratum Corneum Hydration		Transepidermal Water Loss		Skin Surface pH	
		Left Forearm	Right Shin	Left Forearm	Right Shin	Left Forearm	Right Shin
IL-1α	Males	***R* = *-0.2581, p* = *0.0185***	*R* = -0.02294, NS	*R* = -0.1980, NS	*R* = 0.1682, NS	*R* = 0.04047, NS	*R* = -0.1034, NS
	Females	***R* = *-0.2363, p* = *0.0018***	***R* = *-0.1767, p* = *0.0204***	*R* = 0.03520, NS	*R* = 0.06082, NS	*R* = 0.06915, NS	*R* = 0.02276, NS
	Overall	***R* = *-0.2300, p* = *0.002***	*R* = -0.1102, *p* = 0.0791	*R* = -0.02591, NS	*R* = 0.1041, NS	*R* = 0.04531, NS	*R* = -0.03556, NS
IL-1β	Males	*R* = -0.2094, *p* = 0.0575	*R* = -0.1004, NS	*R* = -0.1208, NS	*R* = 0.1750, NS	*R* = -0.1687, NS	***R* = *-0.2339, p* = *0.0333***
	Females	***R* = *-0.2626, p* = *0.0005***	***R* = *-0.2339, p* = *0.0020***	*R* = 0.01403, NS	*R* = 0.02684, NS	*R* = 0.03418, NS	*R* = 0.02138, NS
	Overall	***R* = *-0.2358, p* = *0.0001***	***R* = *-0.1811, p* = *0.0037***	*R* = -0.01860, NS	*R* = 0.08090, NS	*R* = -0.03080, NS	*R* = -0.06734, NS
IL-6	Males	*R* = -0.2026, *p* = 0.0662	*R* = -0.09027, NS	*R* = -0.08329, NS	*R* = 0.1757, NS	*R* = -0.05662, NS	*R* = -0.1859, NS
	Females	***R* = *-0.3273, p* < *0.0001***	***R* = *-0.2794, p* = *0.0002***	*R* = 0.07693, NS	*R* = 0.1223, NS	*R* = 0.07651, NS	*R* = 0.02215, NS
	Overall	***R* = *-0.2547, p* < *0.0001***	***R* = *-0.1900, p* = *0.0023***	*R* = 0.02729, NS	***R* = *0.1472, p* = *0.0187***	*R* = 0.009841, NS	*R* = -0.07664, NS
TNFα	Males	*R* = -0.06558, NS	*R* = -0.06368, NS	*R* = 0.01172, NS	*R* = 0.1622, NS	***R* = *-0.2270, p* = *0.0391***	***R* = *-0.2920, p* = *0.0071***
	Females	***R* = *-0.3171, p* < *0.0001***	***R* = *-0.3625, p* < *0.0001***	*R* = 0.02069, NS	*R* = 0.05679, NS	*R* = 0.1079, NS	*R* = 0.09248, NS
	Overall	***R* = *-0.2025, p* = *0.0011***	***R* = *-0.2318, p* = *0.0002***	*R* = 0.01563, NS	*R* = 0.1015, NS	*R* = -0.01750, NS	*R* = -0.06320, NS
IFNγ	Males	***R* = *-0.2608, p* = *0.0173***	*R* = -0.1574, NS	*R* = -0.1624, NS	*R* = 0.1696, NS	*R* = -0.05261, NS	*R* = -0.1110, NS
	Females	***R* = *-0.1872, p* = *0.0139***	*R* = -0.1431, *p* = 0.0611	*R* = -0.05529, NS	*R* = 0.0007165, NS	*R* = 0.005025, NS	*R* = 0.001154, NS
	Overall	***R* = *-0.2050, p* = *0.001***	***R* = *-0.1449, p* = *0.0206***	*R* = -0.07432, NS	*R* = 0.04781, NS	*R* = 0.002921, NS	*R* = -0.02499, NS
**Age- and Sex-Adjusted Correlation**							
IL-1α		***R* = *-0.269, p* < *0.0001***	***R* = *-0.163, p* = *0.009***	*R* = -0.035, *p* = 0.575	*R* = 0.087, *p* = 0.166	*R* = 0.063, *p* = 0.315	*R* = -0.004, *p* = 0.944
IL-1β		***R* = *-0.244, p* < *0.0001***	***R* = *-0.189, p* = *0.003***	*R* = -0.016, *p* = 0.798	*R* = 0.084, *p* = 0.185	*R* = -0.027, *p* = 0.664	*R* = -0.068, *p* = 0.283
IL-6		***R* = *-0.287, p* < *0.0001***	***R* = *-0.227, p* < *0.0001***	*R* = 0.027, *p* = 0.672	*R* = 0.140, *p* = 0.026	*R* = 0.031, *p* = 0.622	*R* = -0.051, *p* = 0.416
TNFα		***R* = *-0.191, p* = *0.002***	***R* = *-0.195, p* = *0.002***	*R* = 0.035, *p* = 0.577	***R* = *0.127, p* = *0.044***	*R* = -0.013, *p* = 0.842	*R* = -0.082, *p* = 0.192
IFNγ		***R* = *-0.198, p* = *0.002***	***R* = *-0.133, p* = *0.034***	*R* = -0.070, *p* = 0.268	*R* = 0.057, *p* = 0.363	*R* = -0.009, *p* = 0.888	*R* = -0.040, *p* = 0.530

Previous studies have demonstrated a likely pathogenic role for epidermal dysfunction in inflammaging [4, 5]. Although epidermal permeability barrier recovery is delayed, basal transepidermal water loss rates do not differ significantly between aged (> 60 years old) and young human skin (21–30 years old) [10]. Accordingly, transepidermal water loss rates did not correlate with elevations in serum cytokine levels (except TNFa). Likewise, age-associated elevations in skin surface pH did not correlate with serum cytokine levels, even though skin surface pH levels are 0.9 units higher in aged vs. young human skin [10], suggesting that increases in skin surface pH alone do not suffice to provoke inflammaging. However, both permeability barrier disruption and prolonged elevations in skin surface pH can exacerbate cutaneous inflammation [7], resulting in an increase in circulating levels of cytokines, which can be amplified, for example, by scratching. Importantly, the present study revealed that reductions in stratum corneum hydration inversely correlate with elevations in serum cytokine levels, suggesting that inflammaging could be ascribed, in part, to aging-associated decreases in stratum corneum hydration levels.

Previous studies showed that reduced stratum corneum hydration levels increase cytokine release, inflammatory infiltration, and mast cell degranulation in the skin [11]. Conversely, topical applications of emollients can alleviate cutaneous inflammation in skin conditions characterized by low stratum corneum hydration [8, 11]. Sustained low stratum corneum hydration can induce persistent cutaneous inflammation, eventually leading to elevations in circulating levels of proinflammatory cytokines [3]. Accordingly, improvement in stratum corneum hydration with topical emollients lowers serum cytokine levels in both aged humans and mice [4, 5]. Because improvements in epidermal functions, including increased stratum corneum hydration levels, decrease circulating levels of proinflammatory cytokines in both mice and humans, lower stratum corneum hydration levels are unlikely to be caused by elevated proinflammatory cytokines. Instead, reduced stratum corneum hydration likely contributes to inflammaging. Accordingly, a recent pilot clinical trial demonstrated that improvements in stratum corneum hydration attenuate the progression of cognitive impairment, an inflammaging-associated disorder in the aged humans [6].

Collectively, these results indicate that stratum corneum hydration levels, rather than alterations in either transepidermal water loss rates or skin surface pH, inversely correlate with elevated serum cytokine levels in the aged humans. Although our pilot study demonstrated the potential benefits of improving stratum corneum hydration in the mitigation of cognitive impairment [6], further studies are warranted to ascertain whether improvements in stratum corneum hydration can benefit other inflammaging-associated conditions.

## Data Availability

Data are available upon reasonable request.

## Abbreviations

BMI: Body mass index
CI: Confidence interval
IFNγ: Interferon gamma
IL-1a: Interleukin-1alpha
IL-1b: Interleukin-1beta
TNFα: Tumor necrosis factor alpha
TEWL: Transepidermal water loss rate
